# Non-codon Optimized PiggyBac Transposase Induces Developmental Brain Aberrations: A Call for *in vivo* Analysis

**DOI:** 10.3389/fcell.2021.698002

**Published:** 2021-08-03

**Authors:** Franziska Vierl, Manpreet Kaur, Magdalena Götz

**Affiliations:** ^1^Institute for Stem Cell Research, Helmholtz Zentrum München, Munich, Germany; ^2^Physiological Genomics, Biomedical Center, Ludwig-Maximilians-Universität, Munich, Germany; ^3^Graduate School of Systemic Neurosciences, Ludwig-Maximilians-Universität, Munich, Germany; ^4^SyNergy, Munich Cluster for Systems Neurology, Ludwig-Maximilians-Universität, Munich, Germany

**Keywords:** genome integration, transposon, viral vectors, neural stem cells, cortex development, gyrification

## Abstract

In this perspective article, we briefly review tools for stable gain-of-function expression to explore key fate determinants in embryonic brain development. As the piggyBac transposon system has the highest insert size, a seamless integration of the transposed sequence into the host genome, and can be delivered by transfection avoiding viral vectors causing an immune response, we explored its use in the murine developing forebrain. The original piggyBac transposase PBase or the mouse codon-optimized version mPB and the construct to insert, contained in the piggyBac transposon, were introduced by *in utero* electroporation at embryonic day 13 into radial glia, the neural stem cells, in the developing dorsal telencephalon, and analyzed 3 or 5 days later. When using PBase, we observed an increase in basal progenitor cells, often accompanied by folding aberrations. These effects were considerably ameliorated when using the piggyBac plasmid together with mPB. While size and strength of the electroporated region was not correlated to the aberrations, integration was essential and the positive correlation to the insert size implicates the frequency of transposition as a possible mechanism. We discuss this in light of the increase in transposing endogenous viral vectors during mammalian phylogeny and their role in neurogenesis and radial glial cells. Most importantly, we aim to alert the users of this system to the phenotypes caused by non-codon optimized PBase application *in vivo*.

## Introduction

Much has been learnt about fate determinants in development during neurogenesis and gliogenesis from candidate approaches, natural gene mutations and genome-wide expression analyses. To elucidate their function, and subsequently use them e.g., for direct neuronal reprogramming or therapeutic approaches, we need reliable tools for the long-term manipulation of gene expression, e.g., by seamless genomic integration. Tools for this are still relatively limited and mostly rely on viral vectors. The piggyBac transposon system allows genomic integration and long-term expression of genes of interest, providing a viable alternative as described below. These approaches are now timely to review and consider for specific pitfalls, due to the immense power of their potential for therapeutic use, e.g., by direct neuronal reprogramming *in vivo* (Götz and Bocchi, 2021). This applies especially as the CRISPR/Cas technology now facilitates multiplexing, targeting double-digit numbers of genes without problem (Breunig et al., 2018, 2021). This will help understanding the role of transcriptional networks in development, or to manipulate cell fate in a fine-tuned manner—e.g., to achieve correct subtype specification of glia or neurons in reprogramming—but needs to be delivered and integrated in a safe, yet reliable manner.

An important consideration in the use of integrating constructs is the similarity to endogenous transposable elements (TE), which play an important role in development. In humans, these make up the majority of the genome, with up to 69% suggested to be TE (de Koning et al., 2011; Ferrari et al., 2021). In the developing brain, the mobilization and (re-)integration of TE, especially of the endogenous retroviral LINE-1 elements, is linked to the development of neuronal subtype diversity and genomic mosaicism, and dysregulation may lead to developmental abnormalities (see e.g., Bodea et al., 2018; Misiak et al., 2019).

We will therefore review specific approaches to genome editing with an emphasis on gain-of-function and highlight a not yet reported and hitherto not yet fully explained artifact using the piggyBac transposon system *in vivo*.

## Tools for Stable Expression of Genes of Interest

### Viral Vectors for Long-Term Gene Expression

**FIGURE 1:**
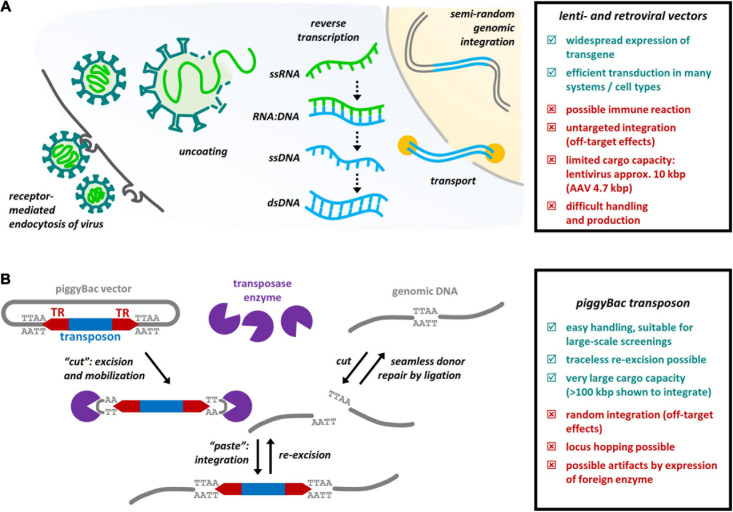
Common methods of genome editing in neural stem cells *in vivo*. **(A)** Retro- or lentiviral transduction. The virus is introduced by receptor-mediated endocytosis depending on the pseudotype of the capsid, which is then degraded to uncoat the viral genome. This is retrotranscribed from ssRNA to dsDNA, transported to the cell’s nucleus and integrated into the genome, with preferred integration sites dependent on the type of virus. **(B)** Integration of the piggyBac transposon. In the presence of its specific transposase, the piggyBac transposon can be integrated to the genome in a “cut-and-paste” manner. TTAA motifs in the terminal repeats (TR) regions are recognized by the transposase which excises and mobilizes the transposon. The genomic DNA is cut at TTAA sites and the transposon is integrated by ligation. In the same way, the transposon can be re-excised, with a seamless re-ligation repairing the host’s donor site.

Viral vectors are a classical tool for the long-term manipulation of gene expression. Especially Retrovirus (RV) and Lentivirus (LV) are often used both *in vitro* and *in vivo*, e.g., to elucidate the function of candidate genes in embryonic development (Artegiani and Calegari, 2013), or in direct neuronal reprogramming approaches (Gascón et al., 2016; Herrero-Navarro et al., 2021; Russo et al., 2021). A big advantage of viral vectors is the ability to target specific cell types of the brain by pseudotyping the capsid (Buffo et al., 2008; Mattugini et al., 2019). Both virus types achieve long-term gene expression by integrating their reverse-transcribed RNA genome into the host genome as DNA (Figure 1A) at semi-random sites, LV preferring active transcription units, while RV favor enhancers and regulatory sites (Schröder et al., 2002). This semi-random integration has the disadvantage of potential off-target effects and the introduction of mutations. While expression systems have been optimized to avoid gene silencing (Pfeifer et al., 2002) and reduce the immune response (Piras et al., 2017; Russo et al., 2021), the limited packaging capacity and the immune reactions still elicited by these viral vectors and their pseudotypes (Mattugini et al., 2019) remain disadvantageous.

For translational therapeutic approaches, adeno-associated virus (AAV) is better suited due to the limited immune response (see e.g., Mattugini et al., 2019; Rittiner et al., 2020). A multitude of serotypes allow targeting specific cell types even via non-invasive, e.g., intravenous, application routes (Haggerty et al., 2020; Nectow and Nestler, 2020; Rittiner et al., 2020). While allowing long-term stable gene expression depending on the cell type, these vectors stay episomal, i.e., do not integrate into the host genome, but may be used to deliver constructs that are themselves able to integrate (such as transposon systems) or to permanently edit the host genome (e.g., CRISPR/Cas9). However, their biggest disadvantage is the small packaging size of less than 5 kBp (Rittiner et al., 2020), and although it can be overcome by splitting some proteins, e.g., Cas9, into two parts (Truong et al., 2015), it generally limits the applicability of these vectors.

### Transposable Elements for Widespread Genomic Integration

The piggyBac system has been discovered as a transposable element decades ago, initially in insect cells (Cary et al., 1989), and was used to generate transgenic vertebrate models with stable inheritance (Ding et al., 2005). A huge advantage is the ability to accommodate large constructs, with sequences of 100 kBp reported to successfully integrate in the genome (Li et al., 2011). There seems to be no bias for integration into specific chromosomes, but a strong preference to integrate into accessible chromatin and highly transcribed genes (Elick et al., 1996; Ding et al., 2005; Wang et al., 2008; Li et al., 2013; Yoshida et al., 2017).

The piggyBac transposon is characterized by terminal repeats ending in TTAA that are necessary for successful transposition (Figure 1B; Cary et al., 1989; Li et al., 2005). The construct to insert, i.e., the transposon, by itself shows little integration, but when paired with its specific transposase even in low amounts, integration is very efficient (Ding et al., 2005; Cadinanos and Bradley, 2007; Wang et al., 2008). In mammalian cells, as in its native system, the piggyBac transposon integrates into the host genome exclusively at TTAA sites (Figure 1B), with the specific transposase enzyme nicking the DNA and inducing transient double strand breaks which are repaired not by DNA synthesis, but rather ligation of the ends. This leads to seamless integration and traceless excision of the transposon (Mitra et al., 2008; Chen et al., 2020).

Due to efficient cloning strategies, the piggyBac system has readily lent itself for screenings *in vivo* (Xu et al., 2017), and the possibility of removing the transgene without a footprint by transposase-mediated excision (Woltjen et al., 2009; Behringer et al., 2017) is promising for translational approaches. The application of the piggyBac transposon for *in vivo* studies has been further encouraged by the development of improved transposase enzymes that, based on the original PBase, have been optimized for their use e.g., in mammalian systems. A mouse-codon optimized version of the enzyme, mPB, has been shown to elicit a 20-fold higher transposition activity in mouse ES cells as compared to the non-optimized PBase, likely due to higher expression levels following the facilitation of the translation process (Cadinanos and Bradley, 2007). Further optimization yielded an even more efficient enzyme, the hyperactive transposase hyPBase, by introducing point mutations in the sequence of mPB (Yusa et al., 2011).

## The Use of piggyBac Transposon in the Developing Brain *in vivo*—A Call for Codon Optimization

Given the advantages of the piggyBac transposon system, we tested different transposases for gene manipulation in the developing cortex. For plasmid DNA, such as the piggyBac transposon, a favored delivery method is *in utero* electroporation (IUE), often performed during mid-gestation between E12 and E15 (Saito, 2006; Meyer-Dilhet and Courchet, 2020).

**FIGURE 2:**
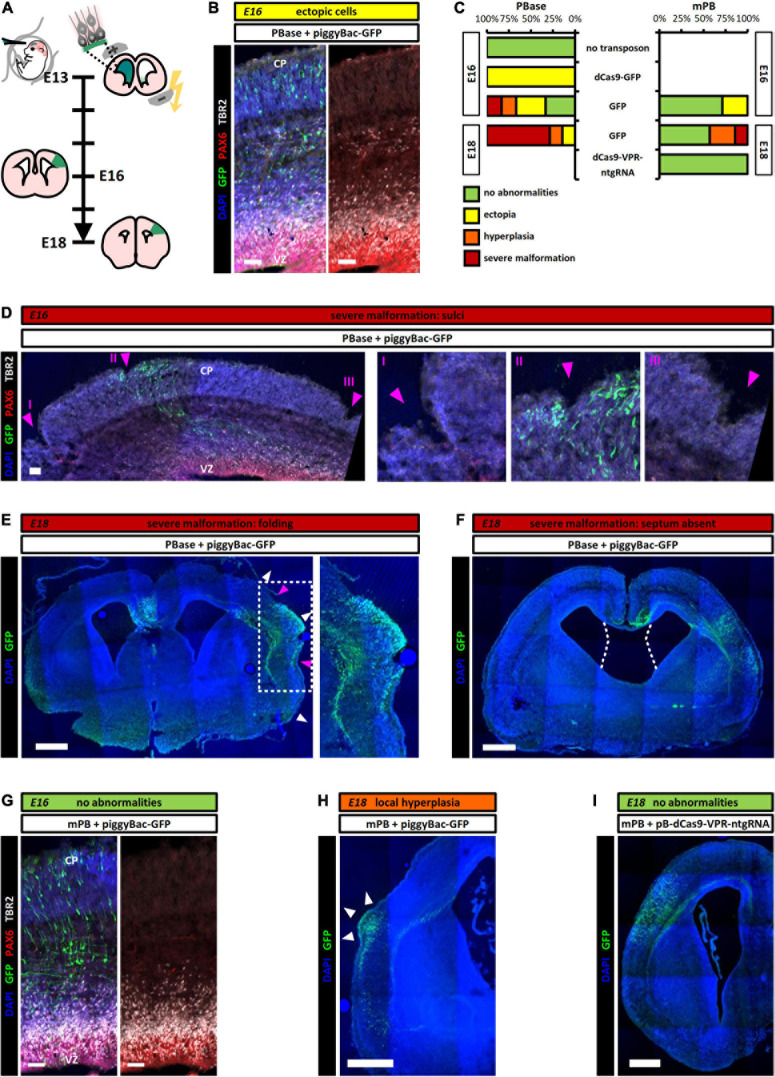
Unexpected abnormalities in cortex development upon electroporation of PBase, but not mPB. **(A)** Experimental schedule. piggyBac transposon plasmids as indicated in Supplementary Figure 1 were delivered into the cortex by IUE at E13 and brains were analyzed at E16 or E18. **(B)** At E16 (3 days post-IUE), transposition of piggyBac-GFP by the original piggyBac transposase PBase leads to different phenotypes, such as an accumulation of ectopic PAX6^+^/TBR2^+^ cells in the intermediate zone of the cerebral cortex (VZ = ventricular zone, CP = cortical plate; scale bar: 50 μm). **(C)** Quantification of the different phenotypes at time points indicated in the graphs. mPB leads to a lower penetrance and milder phenotypes, while PBase causes strong developmental aberrations depending on the co-electroporated transposon. Shorter transposons lead to an exacerbated effect, no abnormalities develop in the absence of a transposon. *n* = *6* for PBase/no transposon (E16), PBase + dCas9-GFP (E16), and PBase + GFP (E16); *n* = *7* for mPB + GFP (E16), PBase + GFP (E18), and mPB + GFP (E18); *n* = *4* for mPB + dCas9-VPR-ntgRNA (E18). **(D)** Example of severe developmental aberration caused by PBase transposition at E16, here leading to the formation of sulcus-like structures at and further away from the electroporation site (Scale bar: 50 μm). **(E)** At E18 (5 days post-IUE), the phenotype of PBase-mediated transposition of piggyBac-GFP is exacerbated, with severe malformation mostly manifesting in folding of the cortex at and further away from the electroporation site (Scale bar: 500 μm). **(F)** Another example of severe malformation by PBase IUE is lack of the medial region (the septum) normally separating the lateral ventricles (Scale bar: 500 μm). **(G)** At E16, IUE of mPB mostly led to a normal phenotype with no ectopic cells (Scale bar: 50 μm). **(H)** Some animals showed developmental abnormalities at E18 after mPB IUE, e.g., local hyperplasia with increased cortical thickness at the electroporation site (Scale bar: 500 μm). **(I)** With mPB-mediated integration of the largest transposon (piggyBac-dCas9-VPR-ntgRNA), no phenotypes were observed (Scale bar: 500 μm).

With the aim of later activating endogenous gene expression, we first administered the original transposase enzyme PBase (Supplementary Figures 1A,B) and a piggyBac transposon containing a fusion protein of enzymatically deactivated Cas9 and GFP (dCas9-GFP, Supplementary Figure 1B) into radial glia by IUE during mid-neurogenesis at E13 (Figure 2A). After 3 days, all of the brains showed ectopia of cells expressing the stem cell marker PAX6 and the basal progenitor (BP) marker TBR2 (Figure 2C). These markers are normally found in a distinctive band in the ventricular and subventricular zone, respectively (Götz et al., 1998; Englund et al., 2005).

To test if the observed phenotype may be caused by the expression of the foreign protein dCas9, we repeated the experiment with a simpler piggyBac transposon containing only a GFP reporter (Supplementary Figure 1B). We again observed developmental abnormalities in the electroporated brains after 3 days (Figures 2B,C). However, the variability here was much higher, ranging from some brains exhibiting no abnormalities, over the previously observed PAX6^+^/TBR2^+^ ectopia, to one embryo exhibiting severe malformations, where the cortex showed several sulcus-like indentations. We noted also a non-cell autonomous phenotype, as the folds were found not only at the electroporation site, but also at more distant positions (Figure 2D).

We then introduced a plasmid only carrying the GFP reporter, lacking the terminal repeats recognized by the transposase enzyme and thus without the possibility to integrate genomically (pCAG-IRES-GFP, Supplementary Figure 1B). In this case, no aberrant phenotype was observed (Figure 2C); a significant difference from the effects elicited by both piggyBac constructs (*p*_adj_ = 0.026 for piggyBac-GFP and *p*_adj_ = 0.005 for piggyBac-dCas9-GFP; Supplementary Table 1). Thus, the observed aberrations are dependent on the integration and transposition activity of the piggyBac system.

To determine if the phenotypes may be transient, we examined the brains later at E18, 5 days after IUE. Now all PBase-expressing brains showed an aberrant and in most cases exacerbated phenotype with anatomical malformations (Figure 2C). These could be distinguished into two levels of severity, with some cortices only showing localized hyperplasia at the electroporation site, whereas others exhibited more severe and widespread malformations, such as folded gyrus- and sulcus-like structures (Figure 2E), and, in one case, no septum with fusion of the lateral ventricles (Figure 2F).

As the PBase enzyme originates from insect cells (Cary et al., 1989), the sequence and codon usage is not adapted to the translation machinery in murine cells. The use of rare codons may influence the ribosomal translation speed and lead to ribosome collisions, a ribotoxic response (RTR) (Wu et al., 2020), and subsequently an unfolded protein stress response (UPR) (Cao and Kaufman, 2012). We thus exchanged the transposase PBase for mPB (Supplementary Figures 1A,B), the mouse codon optimized version of the same enzyme (Cadinanos and Bradley, 2007). This encodes the same amino acid sequence, generating an identical enzyme, but has an altered nucleotide sequence better suited to the murine translation machinery. When expressed together with piggyBac-GFP, only few brains exhibited PAX6^+^/TBR2^+^ ectopia at E16 and most brains did not show any abnormalities (Figures 2C,G).

At E18, we again observed a striking difference between the two versions of the transposase enzyme: Upon IUE of PBase, all brains showed aberrations, in most cases severe, while the phenotype of mPB was considerably milder and observed less frequently (Figures 2C,H), showing a significantly different overall phenotype severity between both enzymes (*p* = 0.017, Supplementary Table 1). Indeed, a piggyBac plasmid containing a dCas9-VPR fusion protein as well as a non-targeted gRNA (ntgRNA) and a GFP reporter (Supplementary Figure 1B), did not elicit any abnormalities 5 days after IUE, when combined with mPB (Figures 2C,I). Thus, we strongly recommend to use the codon-optimized mPB to avoid developmental aberrations.

### Exploring the Cause of Developmental Defects Caused by PBase Application

The above results imply a striking difference between the effects of the codon optimized and the non-codon optimized version of the same enzyme. Both proteins have the same amino acid sequence, but mPB has been found to enable an integration and transposition activity up to 20 times higher than PBase, likely due to a higher expression level in otherwise identical systems (Cadinanos and Bradley, 2007). To test if this is also the case in the developing cortex, we measured the size and intensity of IUE at E18 for the mPB and PBase constructs combined with piggyBac-GFP. There was no detectable difference in the size (Supplementary Figure 2A) or the GFP intensity (Supplementary Figure 2B) of the IUEs between the different transposases, and also no correlation between larger or stronger IUEs and phenotypic alterations.

Next, we asked if the difference in the translation of the transposase enzymes, which is optimized for mPB and suboptimal for PBase due to different codon usage (Cadinanos and Bradley, 2007), may be relevant and elicit differences in the RTR, UPR or DNA damage and double-strand breaks (Ciccia and Elledge, 2010). To explore this, we transfected the different transposase constructs with different piggyBac transposons into adherent neural progenitor cells derived from embryonic cortex (Pollard et al., 2006), but found no upregulation in marker genes for either of these pathways using qRT-PCR (Supplementary Figure 3). Despite PBase being expressed under a stronger promoter (pCAG vs. pCMV for mPB; Supplementary Figure 1B), when measuring transposase mRNA levels, we found a considerably higher expression of mPB than PBase (Supplementary Figure 3B) as suggested previously for expression under the same promoters (Cadinanos and Bradley, 2007). Thus, the promoter seems to have little effect on expression, and despite higher expression levels, mPB causes fewer developmental aberrations.

## Discussion

After reviewing different methods for stable gain-of-function expression systems, we show here an unexpected artifact of the piggyBac transposon system in the developing cortex. Adverse developmental effects clearly depended on the integration of the constructs, suggesting that integration and/or transposition is important. In this regard it is interesting to note that there seems to be a connection between the size of the transposon, ranging between 3.0 and 9.6 kBp, and the severity of the phenotype, as smaller transposons elicited a more severe phenotype than larger ones (Figure 2C and Supplementary Figure 1B). It has been shown that the size of the transposon influences the rate of transposition, which starts decreasing considerably around 9 kBp (Ding et al., 2005). This may suggest that smaller constructs with potentially higher transposition rate are more prone to cause the observed abnormalities. Notably, the expression level of the transposase enzymes (Supplementary Figure 3B) seems not to affect the phenotype severity, possibly due to a saturated ratio of constantly expressed transposase enzyme to the limited number of available transposons. Once bound to the enzyme, only the length of the transposon may influence the speed or efficiency (and thereby the frequency) of the transposition process.

“Locus hopping” in the continued presence of the transposase enzyme may cause problems due to its preference for transcriptionally active sites (Ding et al., 2005; Li et al., 2013), which may disrupt their expression and alter transcription networks, the likelihood of which increases with the transposition activity and each excision and re-integration event. We would therefore suggest to develop mPB constructs that self-inactivate after integration of the transposon has been achieved, to avoid continued transposition events. Additionally, the link to endogenous transposition events has to be considered: While it has been shown that piggyBac transposase is unable to mobilize endogenous, PB-like transposons in mouse or human cells (Yusa et al., 2011; Saha et al., 2015), an endogenous human transposase highly expressed in neurons, PGDB5, has been suggested to potentially mobilize piggyBac transposons (Henssen et al., 2015).

It is intriguing that specifically the generation of PAX6^+^ and/or TBR2^+^ BPs and folding of the brain were affected, as these cells comprise an important glial subtype, the basal radial glial cells, and are particularly frequent in species with folded cortices (Borrell and Götz, 2014). Interestingly, in the developing gyrified cortex, they occur in a blockwise manner, with folds formed at the sites of expanded basal subventricular zone where basal radial glial cells are most frequent (Borrell and Götz, 2014). This organization can be mimicked even in the normally smooth murine cortex e.g., by locally lowering the levels of TRNP1, a protein involved in nuclear phase transition (Esgleas et al., 2020). Depletion of the protein, which in murine cortex is normally expressed in a salt-and-pepper fashion, in a specific region by IUE artificially causes blockwise expression, generating a region of expanded subventricular zone and basal radial glial cells, and accordingly induces cortical folding (Stahl et al., 2013; Borrell and Götz, 2014). We do not know if the ectopic BPs and folding that we observed upon PBase-mediated transposition after IUE activate a natural endogenous program, but it is intriguing to see how readily BP number and position may be influenced.

As noted above, the phenotype we observed was not strictly cell autonomous, as was also the case in the TRNP1 manipulations (Stahl et al., 2013; Esgleas et al., 2020) and other means to expand BP numbers (Rash et al., 2013; Xie et al., 2019). It will thus be very important to understand, if—and which—cell surface molecules or secreted signals may be regulated by these manipulations. Given the important communication *via* secreted vesicles in the cerebrospinal fluid (Marzesco et al., 2005; Morton and Feliciano, 2016), these may be involved in non-cell autonomous communication within the cortex tissue.

While the human cortex is naturally gyrified, the developmental disorder polymicrogyria (PMG) is characterized by the generation of additional, aberrant gyri in the neocortex. While PMG is commonly considered a neuronal migration disorder (Romero et al., 2018), several studies have also suggested causative progenitor and proliferation aberrations. Mutations in *cyclin D2* were linked to a cortical malformation syndrome including PMG in patients, and IUE of these mutated genes caused an increased proliferation of progenitors and impaired cell-cycle exit in the developing mouse cortex (Mirzaa et al., 2014). In addition, PMG patients with mutations in the *Pax6* as well as *Tbr2* genes have been identified (Mitchell et al., 2003; Baala et al., 2007), which may relate to the data presented here with ectopic PAX6^+^ or TBR2^+^ cells and additional folds in this study.

While it is not immediately evident why the transposition would cause an increase in BPs, it is intriguing to consider that the naturally occurring transposition by recently integrated endogenous retroviruses (ERVs) may play a similar role, as their proportion hugely increases in the mammalian and especially the primate genome, correlating to cortex size and gyrification (Linker et al., 2017). Indeed, ERVs can regulate the expression of neighboring genes (Fasching et al., 2015; Brattas et al., 2017; Jonsson et al., 2019, 2020; Petri et al., 2019) and may thus have evolved as a novel gene regulatory machinery involved in enlarging and folding brain regions (Ferrari et al., 2021).

It may thus be interesting to follow up the artifact caused by the piggyBac transposon system mechanistically, by controlling transposition events and the generation of BPs. For the use of the piggyBac transposon system it is important to use codon optimized mPB and develop transposase removal strategies to minimize transposition after initial integration. Foremost, however, it is important to be aware of this pitfall, which has so far not been reported, and how to optimize the system as detailed here.

## Data Availability Statement

The raw data supporting the conclusions of this article will be made available by the authors, without undue reservation.

## Ethics Statement

The animal study was reviewed and approved by the Government of Upper Bavaria.

## Author Contributions

MG and FV conceived, conceptualized the project, and wrote the perspective. FV performed all experiments and analyses shown. MK aided in preliminary experiments revealing the phenotype difference between transposase enzymes. All authors contributed to the article and approved the submitted version.

## Conflict of Interest

The authors declare that the research was conducted in the absence of any commercial or financial relationships that could be construed as a potential conflict of interest.

## Publisher’s Note

All claims expressed in this article are solely those of the authors and do not necessarily represent those of their affiliated organizations, or those of the publisher, the editors and the reviewers. Any product that may be evaluated in this article, or claim that may be made by its manufacturer, is not guaranteed or endorsed by the publisher.
