# Supplementary material for: Non-codon Optimized PiggyBac Transposase Induces Developmental Brain Aberrations: A Call for in vivo Analysis
Source: Front Cell Dev Biol. 2021 Aug 3;9:698002. doi: 10.3389/fcell.2021.698002 (PMC8369470; doi:10.3389/fcell.2021.698002)
Supplement: Supplementary file 1 [file Data_Sheet_1.PDF]

Supplementary Material

1 Supplementary Data

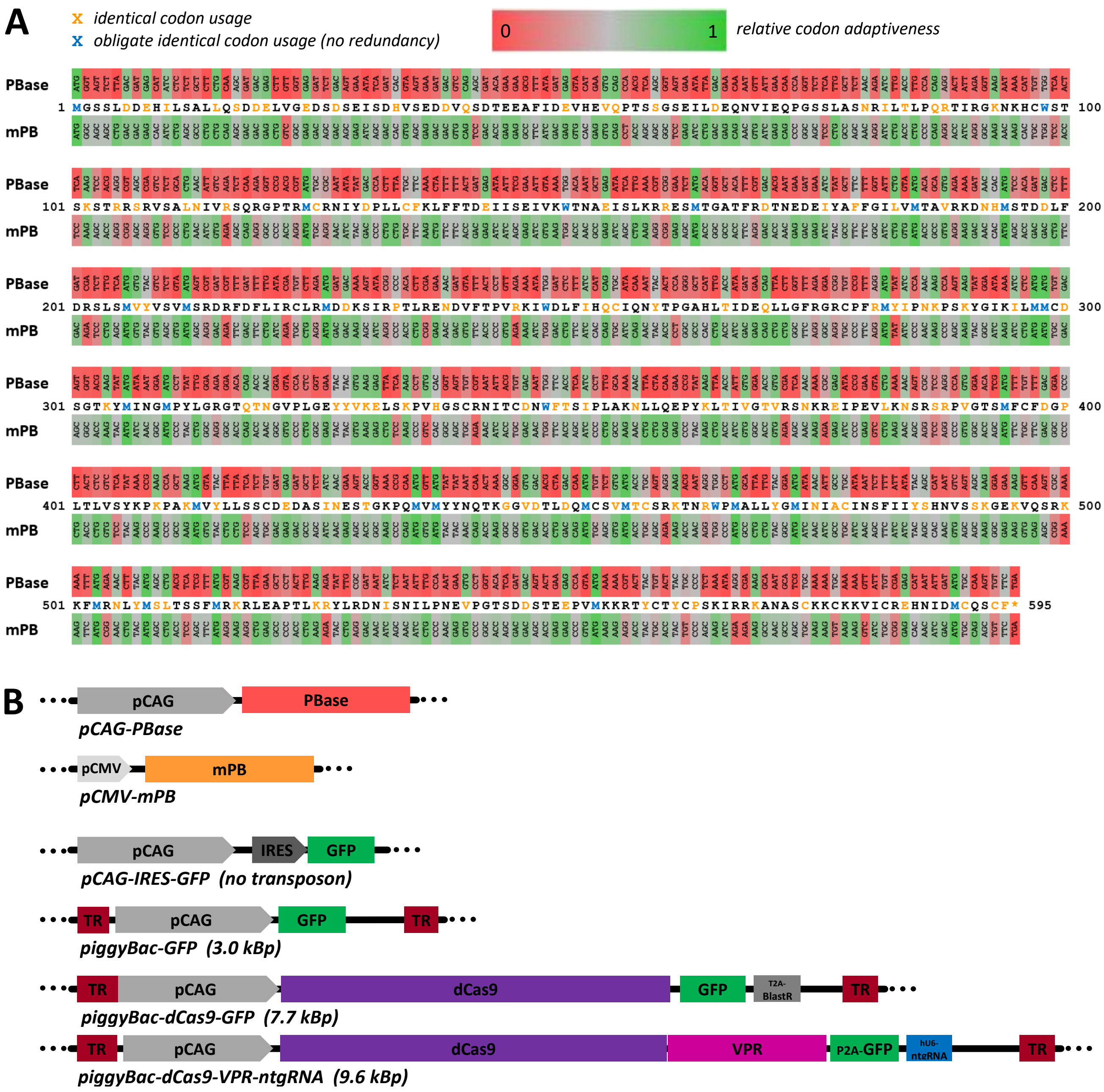

Figure S1. Sequences and plasmid constructs. (A) Comparison of codon usage and adaptiveness to the murine translational machinery between mPB and PBase. Both have the same protein sequence, but differ considerably in their nucleotide sequence (see also Cadinanos and Bradley, 2007; Wang et al., 2008). **(B)** Schematic representation of insert constructs for the plasmids used in this study. Transposon size between terminal repeats (TR) is noted, where applicable.

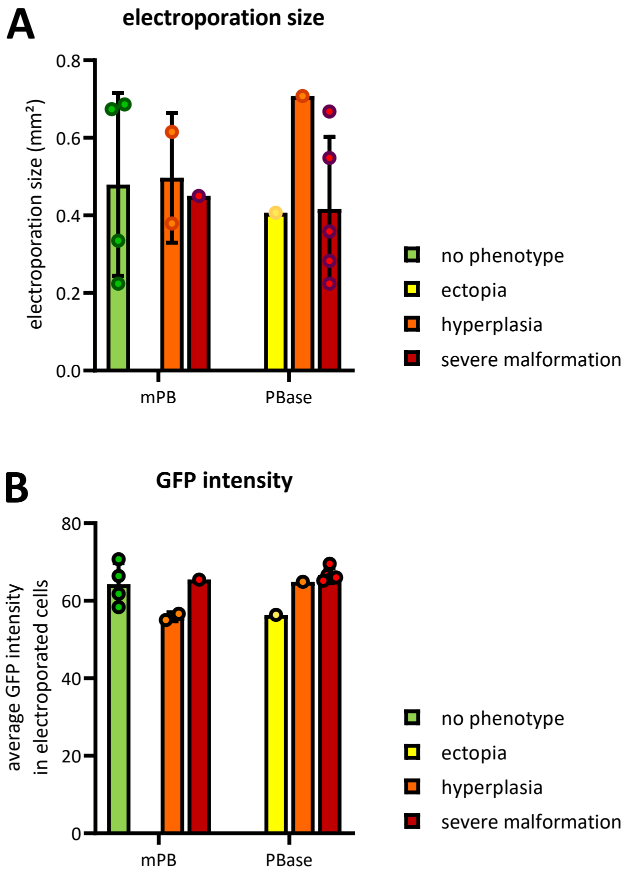

Figure S2. Quantification of electroporation size and intensity at E18. Quantifications were performed of all E18 animals with piggyBac-GFP and either transposase (*n=7* for both). **(A)** Electroporation size was measured as the area that contained GFP^{+} somata. No correlation to the transposase version or severity of the phenotype was observed. **(B)** The average intensity of the GFP signal was quantified as a proxy for the transposon copy number. No correlation to the transposase version or the severity of the phenotype was observed.

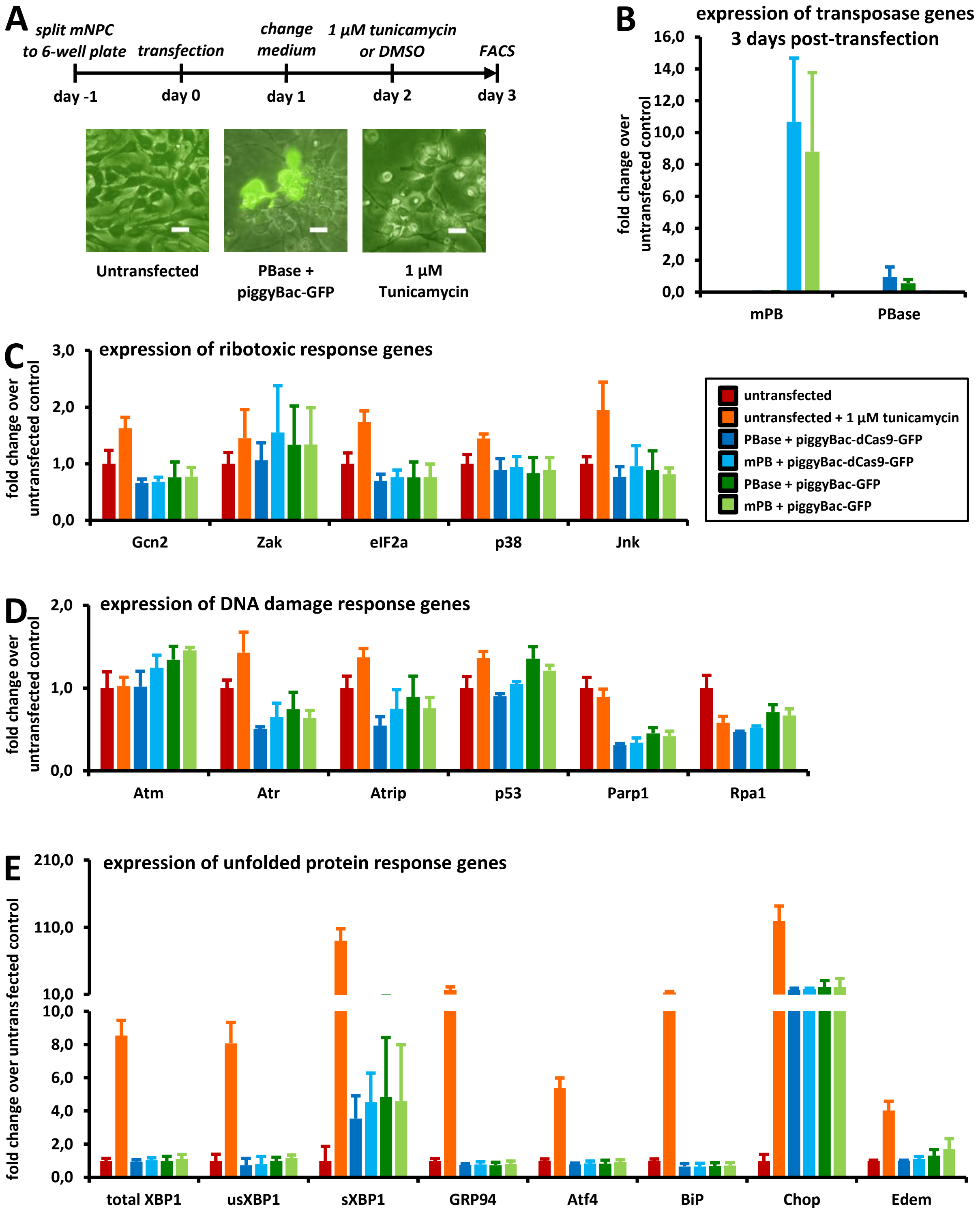

Figure S3. Cellular response to transfection of piggyBac transposon system in adherent neural stem cells. A. Experimental paradigm with sorting of cells labelled three days after transfection. Tunicamycin, a component that elicits a strong unfolded protein response (Godin et al., 2016), was used as positive control. Representative images (brightfield and endogenous GFP signal) were taken before FACS sorting and show a generally increased apoptosis in treated cells. (Scale bars: 20 µm)

B. The expression levels of mPB and PBase were analyzed by qRT-PCR with specific primers for each sequence. Note the higher expression level of mPB than PBase, consistent with previous data in other cells (Cadinanos and Bradley, 2007). **C.** Expression of selected genes involved in the ribotoxic stress response (Wu et al., 2020) were examined by qRT-PCR with specific primers for each sequence. No up-regulation could be detected in any of the conditions. **D.** Expression of selected genes involved in the DNA damage and double strand break response (Ciccia and Elledge, 2010) were examined by qRT-PCR with specific primers for each sequence. No up-regulation could be detected in any of the conditions. **E.** Expression of selected genes involved in the unfolded protein response (UPR) (Cao and Kaufman, 2012) were examined by qRT-PCR with specific primers for each sequence. While we observed strongly upregulation in the positive control upon treatment with tunicamycin, the UPR marker spliced XBP1 (sXBP1) was upregulated in all conditions with piggyBac transfection. The apoptosis marker Chop is highly upregulated in all transfection conditions independent of the plasmids, demonstrating the response elicited by transfection itself, not the different piggyBac constructs. Depicted are the mean + S.D. of biological replicates. *n=5*: Untransfected; *n=4*: Untransfected + Tunicamycin, PBase + piggyBac-GFP, mPB + piggyBac-GFP; *n=3*: PBase + piggyBac-dCas9-GFP, mPB + piggyBac-dCas9-GFP.

Table S1: Statistical analysis of transposase-elicited phenotypes. Phenotypes were assigned numerical scores from 0 (no abnormalities) to 3 (severe malformation). Comparisons were made between the different transposase enzymes to determine the effect of codon optimization and between different transposon lengths. Statistical methods and significance are noted for each comparison. *n=6* for PBase / no transposon (E16), PBase + dCas9-GFP (E16), and PBase + GFP (E16); *n=7* for mPB + GFP (E16), PBase + GFP (E18), and mPB + GFP (E18); *n=4* for mPB + dCas9-VPR-ntgRNA (E18).

<table>
<tr><th>effect of codon optimization</th><th>effect of transposon length</th></tr>
<tr><td rowspan="2"><u>PBase + piggyBac-GFP vs. mPB + piggyBac-GFP</u>
Multiple t-test with Holm-Sidak method
• at E16: # (p=0.094)
• at E18: * (p=0.017)</td><td><u>PBase at E16</u>
Kruskal-Wallis ANOVA with Dunn’s multiple comparisons correction
• no transposon vs. piggyBac-GFP: * (p_{adj}=0.026)
• no transposon vs. piggyBac-dCas9-GFP: ** (p_{adj}=0.005)
• piggyBac-GFP vs. piggyBac-dCas9-GFP: not significant</td></tr>
<tr><td><u>mPB at E18</u>
Mann-Whitney test
• piggyBac-GFP vs. piggyBac-dCas9-VPR-ntgRNA: not significant (p=0.236)</td></tr>
</table>

2 Supplemental Methods

2.1 Plasmid constructs

The pCMV-mPB construct was kindly provided by Rebeca Sánchez González (Dept. Molecular, Cellular and Developmental Neurobiology, Instituto Cajal, Madrid, Spain); and the pCAG-PBase construct by Dr. Germán Camargo Ortega (Dept. Biosystems and Engineering, ETH Zürich, Basel, Switzerland). piggyBac-dCas9-GFP was kindly provided by Dr. Anna Köferle (Epigenetic Engineering, ISF, Helmholtz Center München, Germany; now at Boehringer Ingelheim RCV GmbH & Co KG, Vienna, Austria) and piggyBac-GFP by Dr. Miriam Esgleas Izquierdo (Institute of Stem Cell Research, Helmholtz Center München, Germany; now at ViGeneron GmbH, Planegg, Germany). The sequence for the piggyBac-dCas9-VPR-ntgRNA plasmid was designed by Dr. Christopher Breunig (Epigenetic Engineering, Institute of Stem Cell Research, Helmholtz Center München, Germany; now at ISAR Bioscience GmbH, Planegg, Germany).

2.2 Animal husbandry and experiments

Animal experiments were performed in the Core Facility Animal Models of the Biomedical Center, Ludwig-Maximilians-Universität München according to German and European Union guidelines and were approved by the government of Upper Bavaria (ROB). C57BL/6J mice were obtained from Charles River (Sulzfeld, Germany) or bred on site. Animals were housed in individually ventilated cages in a specified pathogen free facility on a 12:12 hour light/dark cycle with access to food and water *ad libitum*. Mice were mated in the afternoon, with a vaginal plug on the following morning considered as E0. In utero electroporation was performed as described previously (Esgleas et al., 2020) with a 1:1 molar ratio of transposase and piggyBac plasmids, and embryonic brains were collected three or five days post-surgery.

2.3 Tissue processing, immunohistochemistry and imaging

Brains were dissected and immediately fixed by submerging in 4 % paraformaldehyde (in PBS) for 7 hours at 4 °C. They were subsequently cryoprotected by incubation in 30 % sucrose (in PBS) overnight at 4 °C. For sectioning, they were embedded in Neg-50™ medium (ThermoFisher) and cut at 25 µm (E16) or 30 µm (E18) thickness on a cryostat.

For immunostaining, the slides were incubated with the primary antibodies in staining solution (PBS / 10 % normalized goat serum / 0.5 % Triton X-100) overnight at 4 °C, followed by secondary antibodies in staining solution for one hour at room temperature. Primary antibodies were used against GFP (chicken, 1:200, Aves Labs, #GFP-1020), PAX6 (mouse, 1:25, Core Facility Monoclonal Antibodies, Helmholtz Center Munich, Germany) and TBR2 (rabbit, 1:200, Abcam, #ab183991). Secondary antibodies of complimentary species, coupled to Alexa fluorophores, were used. DAPI (4',6-diamidin-2-phenylindol, Sigma) was used to visualize the cell nuclei.

Images were taken on a confocal microscope (Zeiss LSM710) using Zen software (Carl Zeiss). Quantification of electroporation area and intensity was performed with ImageJ Fiji. Electroporation size was determined by the cortex area that contained GFP^+ cell bodies. Any GFP intensity considered background signal was excluded by a threshold of 35. A mask for any areas exceeding this intensity value was created and the corresponding area of GFP signal, as well as the average signal intensity, were measured in the original image.

2.4 Statistical analysis of transposase phenotypes

To facilitate the statistical analysis of the qualitative data raised in this study, numerical scores were assigned to the different phenotypic levels as follows: no abnormalities, 0; ectopia, 1; hyperplasia, 2; severe malformation, 3. Statistical tests were performed with these assigned scores to compare different conditions as detailed in Table S1. Statistical analysis was performed using GraphPad Prism v8.3.0 (GraphPad Software, LLC).

2.5 Analysis of codon usage and adaptiveness

Coding nucleotide sequences of transposase enzymes were drawn from respective publications (Cadinanos and Bradley, 2007; Wang et al., 2008) and confirmed by Sanger sequencing. Codon adaptiveness was determined using JCat (www.jcat.de/Grote et al., 2005),with a value of 1 representing optimal adaptation to the murine translational machinery.

2.6 Culture and transfection of adherent neural stem cells

Adherent neural stem cells were kindly provided by Andrea Neuner (Epigenetics Engineering group, Institute of Stem Cell Research, Helmholtz Zentrum München, Germany). Cell were kept in NeuroCult NS-A proliferation medium and supplement (Stem Cell Technologies) with 0.1 % Laminin (Sigma-Aldrich, #L2020), 0.2 % human epidermal growth factor (Stem Cell Technologies, #78006) and 0.1 % human fibroblast growth factor (PeproTech, 100-18B). Cells were passaged at approx. 80 % confluence with Accutase (Gibco, #A1110501) and plated on Poly-D-Lysine coated tissue culture plates. Transfection was performed with Lipofectamine™ 2000 (Invitrogen, #11668) according to the manufacturer's protocol, with a mock transfection performed without plasmid DNA. Cells were treated with 1 µM Tunicamycin (Sigma-Aldrich, #SML1287) or DMSO control 48 hours post-transfection.

2.7 FACS sorting and expression analysis by qRT-PCR

Cells were sorted in a FACSaria IIIu flow cytometer (BD Biosciences) 72 hours post-transfection. Gating strategy included sorting for live cells (forward scatter, FSC / side scatter), single cells (FSC-area / FSC-width) and GFP^+ cells (FSC / 530/30) for transfected samples and GFP^- cells for untransfected cells and mock transfection. Cells were collected and RNA was isolated using the Arcturus PicoPure Kit (Applied Biosystems, #KIT0204).

RNA was reverse transcribed using the Maxima FirstStrand cDNA Synthesis Kit (Thermo Scientific, #K1642) and quantitative real-time PCR was performed on a QuantStudio 6 Flex System (ThermoFisher) using SYBR green reagent mix (Applied Biosystems, #A25742) and custom primers.

3 Supplemental References

Cadinanos, J., and Bradley, A. (2007). Generation of an inducible and optimized piggyBac transposon system. *Nucleic Acids Res* 35(12)**,** e87. doi: 10.1093/nar/gkm446.

Cao, S.S., and Kaufman, R.J. (2012). Unfolded protein response. *Curr Biol* 22(16)**,** R622-626. doi: 10.1016/j.cub.2012.07.004.

Ciccia, A., and Elledge, S.J. (2010). The DNA damage response: making it safe to play with knives. *Mol Cell* 40(2)**,** 179-204. doi: 10.1016/j.molcel.2010.09.019.

Esgleas, M., Falk, S., Forne, I., Thiry, M., Najas, S., Zhang, S., et al. (2020). Trnp1 organizes diverse nuclear membrane-less compartments in neural stem cells. *EMBO J* 39(16)**,** e103373. doi: 10.15252/embj.2019103373.

Godin, J.D., Creppe, C., Laguesse, S., and Nguyen, L. (2016). Emerging Roles for the Unfolded Protein Response in the Developing Nervous System. *Trends Neurosci* 39(6)**,** 394-404. doi: 10.1016/j.tins.2016.04.002.

Grote, A., Hiller, K., Scheer, M., Munch, R., Nortemann, B., Hempel, D.C., et al. (2005). JCat: a novel tool to adapt codon usage of a target gene to its potential expression host. *Nucleic Acids Res* 33(Web Server issue)**,** W526-531. doi: 10.1093/nar/gki376.

Wang, W., Lin, C., Lu, D., Ning, Z., Cox, T., Melvin, D., et al. (2008). Chromosomal transposition of PiggyBac in mouse embryonic stem cells. *Proc Natl Acad Sci U S A* 105(27)**,** 9290-9295. doi: 10.1073/pnas.0801017105.

Wu, C.C., Peterson, A., Zinshteyn, B., Regot, S., and Green, R. (2020). Ribosome Collisions Trigger General Stress Responses to Regulate Cell Fate. *Cell* 182(2)**,** 404-416 e414. doi: 10.1016/j.cell.2020.06.006.
